# Investigation of clinical applicability of deep learning-based virtual contrast-enhanced MRI for nasopharyngeal carcinoma patients

**DOI:** 10.3389/fonc.2026.1859909

**Published:** 2026-08-14

**Authors:** Guangping Zeng, Wen Li, Xue Li, Rui Sun, Saikit Lam, Tianyu Xiong, Ho-Yin Cheung, Ho-Fun Lee, Kar-Ho Lee, Lai-Yin Cheung, Chenbin Liu, Zhou Liu, Xiaping Wei, Jing Cai

**Affiliations:** 1 Department of Radiation Oncology, Jinshazhou Hospital of Guangzhou University of Chinese Medicine, Guangzhou, China; 2 Department of Health Technology and Informatics, The Hong Kong Polytechnic University, Hong Kong, Hong Kong SAR, China; 3 Guangyuan Central Hospital, Guangyuan, Sichuan, China; 4 Chongqing University Cancer Hospital, Chongqing, China; 5 Department of Biomedical Engineering, The Hong Kong Polytechnic University, Hong Kong, Hong Kong SAR, China; 6 Radiotherapy and Oncology Centre, Hong Kong Baptist Hospital, Hong Kong, Hong Kong SAR, China; 7 Department of Clinical Oncology, The University of Hong Kong, Hong Kong, Hong Kong SAR, China; 8 Department of Clinical Oncology, Queen Elizabeth Hospital, Hong Kong, Hong Kong SAR, China; 9 Department of Clinical Oncology, Oncology Center, St. Paul’s Hospital, Hong Kong, Hong Kong SAR, China; 10 Department of Radiology, National Cancer Center/National Clinical Research Center for Cancer/Cancer Hospital & Shenzhen Hospital, Chinese Academy of Medical Sciences and Peking Union Medical College, Shenzhen, Guangdong, China

**Keywords:** clinical applicability, deep learning, nasopharyngeal carcinoma, patient stratification, virtual contrast-enhanced MRI

## Abstract

**Purpose:**

There is a lack of research evaluating the clinical performance of virtual contrast-enhanced MRI (VCE-MRI). This study aims to assess the clinical utility of an established VCE-MRI technique in NPC patients and to establish patient selection criteria.

**Materials and methods:**

We retrospectively collected data from 333 NPC patients across six institutions (2012-2023). VCE-MRI was synthesized from T1-weighted and T2-weighted MRI for each patient using the multimodality-guided synergistic neural network (MMgSN-Net), which was pre-trained on a large cohort of 1682 NPC patients from 14 institutions. Three experienced radiologists independently assessed image quality using a 5-point Likert scale, with scores below 4 deemed clinically unacceptable. The association between assessment results and patient clinical characteristics of corresponding patients (tumor diameter, T-stage, shape, gender, age) was analyzed to stratify the patients suitable for MMgSN-Net-based VCE-MRI.

**Results:**

Clinically acceptability of VCE-MRI significantly decreased with larger tumor diameters (p=0.001), advanced T-stage (T1: 100%, T2: 95.1%, T3: 90.0%, T4: 69.6%; p<0.001), and complex tumor shape (regular: 94.8% vs. complex: 74.0%; p<0.001). Age and gender had no significant impact (p=0.327, 0.773). 13 unacceptable patients were found due to severe VCE-MRI artifacts. VCE-MRI achieved high clinical acceptability in T1/T2 patients (109/114, 95.6%) and T3/T4 patients with regular tumor shapes (115/120, 95.8%). In contrast, acceptability significantly decreased to 73.0% (65/89) for T3/T4 tumors with complex shapes. The T-staging result discordance rate between VCE-MRI and CE-MRI is 8.4%, with overstaging in 15 cases.

**Conclusion:**

MMgSN-Net-based VCE-MRI demonstrates favorable clinical feasibility in selected patient subgroups, T1/T2-stage NPC and T3/T4-stage NPC with regular tumor morphology, potentially reducing GBCAs administration in 67.3% of eligible patients when applying these stratification criteria.

## Introduction

Nasopharyngeal carcinoma (NPC) is a malignancy with high invasive and metastatic potential, is endemic to Southern China and Southeast Asia (1). Magnetic resonance imaging (MRI), particularly contrast-enhanced MRI (CE-MRI) utilizing gadolinium-based contrast agents (GBCAs), is critical for NPC diagnosis, accurate staging (T/N), radiotherapy target delineation, and treatment response assessment (2–4). CE-MRI provides unparalleled soft tissue contrast, essential for delineating tumor extent, detecting deep invasion (e.g., skull base, cranial nerves, parapharyngeal space), and evaluating vascular involvement, making it indispensable for diagnosing and treating NPC (3, 4).

However, recent studies have shown that repeated administrations of GBCAs can lead to gadolinium accumulation in various organs, including the brain, liver, kidneys, skin, and bones, and may cause serious late-stage side effects such as nephrogenic systemic fibrosis (5).

NPC patients require long-term post-treatment surveillance, and repeated contrast-enhanced MRI are routinely performed, leading to a significantly higher risk of cumulative gadolinium exposure than other tumor types (6). Meanwhile, conventional enhanced scanning is contraindicated in patients with renal insufficiency, which further highlights the clinical necessity of VCE-MRI (7). Although the long-term effects of gadolinium deposition are not yet confirmed, growing safety concerns are prompting a more cautious approach. Consequently, minimizing cumulative GBCA exposure has become a significant clinical priority (8).

Deep learning presents a promising approach for reducing GBCA dependence by synthesizing virtual contrast-enhanced MRI (VCE-MRI) from non-contrast sequences (9). Various deep learning architectures, including U-Net, generative adversarial networks (GANs), and convolutional neural networks (CNNs), have demonstrated feasibility in synthesizing VCE-MRI for diverse anatomical regions (10–13). However, these investigations primarily focus on technical validation, employing quantitative metrics (e.g., MAE, MSE, PSNR, SSIM) and limited qualitative assessments to demonstrate image similarity to real CE-MRI.

Despite these technical advances, a critical translation gap persists. Most current VCE-MRI studies take pixel-level quantitative metrics as the core evaluation standard, and few conduct independent clinical assessment by clinicians, particularly under multi-center and multi-device settings. Furthermore, there is no analysis of how specific patient or tumor characteristics influence synthesis quality, and no standardized patient stratification criterion for NPC to guide selective clinical application. This precisely leads to the clinical filling value of this study.

This study comprehensively assessed the clinical utility of a deep learning-based VCE-MRI technique (Multimodality-guided Synergistic Neural Network, MMgSN-Net) within a multi-institutional cohort of NPC patients. We employed expert radiologist assessment using the 5-point Likert scale as the gold standard to determine clinical acceptability of VCE-MRI (14). The core innovation of this work lies in systematically correlating the VCE-MRI quality assessments with detailed patient clinical characteristics, including tumor T-stage, tumor diameter, tumor shape, gender, and age. Through this analysis, we aim to establish patient stratification criteria to identify subgroups best suited for MMgSN-Net-generated VCE-MRI. Specifically, this strategy targets low-risk patients, such as those undergoing follow-up re-examinations or presenting with early-stage lesions, to reduce unnecessary GBCA administration while ensuring diagnostic safety. Data sourced from multiple institutions provides a robust foundation for evaluating broader clinical applicability.

## Materials and methods

This study was approved by the Institutional Review Board of the University of Hong Kong/Hospital Authority Hong Kong West Cluster (HKU/HA HKW IRB, reference number: UW21-412) and the Research Ethics Committee (Kowloon Central/Kowloon East, reference number: KC/KE-18-0085/ER-1). Due to the retrospective nature of this study, patient consent was waived.

### Study population

**Figure 1:**
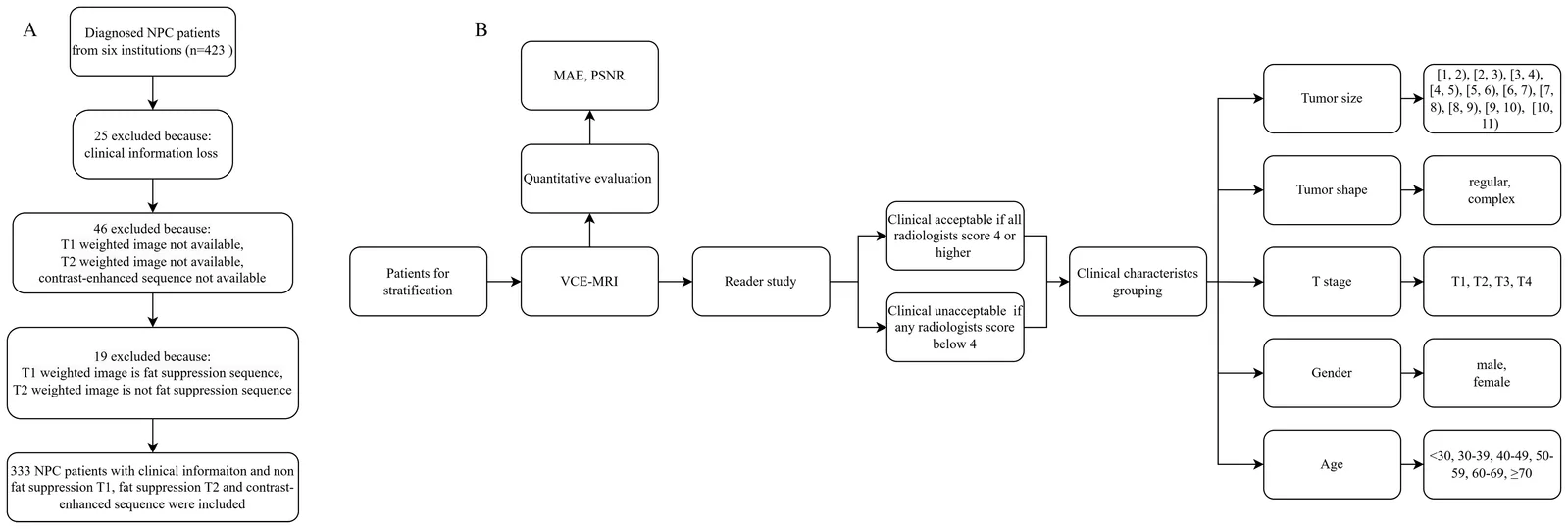
Study flow diagram. **(A)** Study population shows inclusion and exclusion criteria for the NPC MRI scans used in our study. **(B)** VCE-MRI stratification process. NPC, nasopharynx cancer; VCE, virtual contrast-enhanced MRI.

We included 423 diagnosed NPC patients from six medical institutions between 2012 and 2023. We included all NPC examinations consisting of at least a non-fat suppression T1, a fat suppression T2 and a contrast-enhanced sequence; a total of 90 incomplete examinations were excluded. The final number of NPC MRI included in this study was 333 (**Figure 1A**).

### MRI acquisition

MRI sequences for each patient included T1-weighted (T1w), T2-weighted (T2w), and CE-MRI. The number of patients from each institution and scanning protocols are detailed in **Table 1**. Images were acquired at field strengths of 1.5T or 3T. The images were acquired from 13 different scanning devices, including Philips (Prodiva CX, Ingenia, Achieva), Simens (Skyra, Avanto, Biograph_mMR, MAGNETOM Vida), and GE (Signa Voyager, Signa HDxt, SIGNA Explorer, Optima MR450w, SIGNA Pioneer, DISCOVERY MR750w).

**Table 1:** Image counts and scanning parameters of different sites.

Sites	Count	Modality	TR (ms)	TE (ms)	Flip angle	Resolution (mm)
Site 1	21	T1 w	360-600	10.3-14.4	90-150	0.4×0.4×3-0.5×0.5×5
		T2 w	3180-10680	80.5-148.6	90-120	0.3×0.3×3-0.5×0.5×6
		CE-MRI	6.2-8.1	2.5-3.1	12-20	0.5×0.5×1-0.7×0.7×6
Site 2	95	T1 w	6.8-3733	4.4.-86.4	15-180	0.3×0.3×3-1.3×1.3×5
		T2 w	500-12154	47.6-139.1	90-180	0.3×0.3×3-1.3×1.3×5
		CE-MRI	7.3-3149	3.4-99.1	8-180	0.3×0.3×0.8-1.0×1.0×5
Site 3	78	T1 w	NA	NA	NA	0.2×0.2×3-0.8×0.8×5
		T2 w	NA	NA	NA	0.2×0.2×3-0.8×0.8×6
		CE-MRI	NA	NA	NA	0.2×0.2×0.8-1.0×1.0×5
Site 4	91	T1 w	NA	NA	NA	0.2×0.2×2-1.3×1.3×4
		T2 w	NA	NA	NA	0.2×0.2×0.6-1.7×1.7×8
		CE-MRI	NA	NA	NA	0.2×0.2×1.2-1.0×1.0×6
Site 5	18	T1 w	413-747	6.5-18	90-160	0.4×0.4×3.5-0.8×0.8×5.5
		T2 w	2165-4139	74-92	90-150	0.4×0.4×3.5-0.8×0.8×5.5
		CE-MRI	561-849	6.5-18	90-150	0.3×0.3×3.5-0.8×0.8×5.5
Site 6	30	T1 w	434-850	7.1-10.9	111-142	0.4×0.4×4-0.9×0.9×5
		T2 w	3927-11000	80-107	111-142	0.4×0.4×4-0.5×0.5×5
		CE-MRI	401-824	8.2-10.4	111-142	0.4×0.4×4-0.9×0.9×5

NA, not applicable; TR, repetition time; TE, echo time. This table shows the number of patients and the scanning parameters in different sites. The scanning parameters include repetition time (TR), echo time (TE), flip angle and resolution (pixel spacing X * pixel spacing Y * slice thickness). The TR, TE and flip angle parameters of Site 3 and Site 4 were not available.

### VCE-MRI synthesis

The MMgSN-Net, a previously established 2D deep learning network, was employed to synthesize VCE-MRI from T1w and T2w. This model comprises five core modules: multimodality learning module, synergistic guidance system (SGS), self-attention module, multi-level module, and discriminator (11). MMgSN-Net was trained with 1682 patients from 14 institutions. In this study, we included 333 new patients for clinical evaluation.

### Assessment of VCE-MRI clinical applicability

Three radiologists with over five years of clinical experience evaluated the quality of synthetic VCE-MRI, identifying and recording reasons for images deemed unsuitable for clinical use. Radiologists used ITK-SNAP (version 4.0.2) (15) to evaluate the reconstructed 3D volumetric MHA format image data, assigning scores using 5-point Likert scale (16). VCE-MRI was considered clinically acceptable if all three radiologists assigned scores ≥ 4; any score < 4 from three radiologists was deemed clinically unacceptable. The assessment criteria included changes in the tumor and surrounding structures, clarity of anatomical details, boundary clarity of tumor and surrounding structures, presence of artifacts, and tumor and vascular enhancement signals. Scoring details were as follows:

5: Indistinguishable from CE-MRI, excellent diagnostic confidence. 4: Minor differences from CE-MRI, suitable for clinical use. 3: Noticeable degradation, not suitable for clinical use. 2: Severe image degradation or artifact, not suitable for clinical use. 1: Non-diagnostic image quality.

Discrepancies ≥ 2 between the scores prompted consensus adjudication from all 3 radiologists. Additionally, T staging of the CE-MRI and VCE-MRI was independently performed by one experienced radiologist and confirmed by two other radiologists, and then the T-stage result was compared between them.

To determine whether VCE-MRI meets the basic requirements for radiotherapy target delineation, two radiation oncologists (with 10 years of experience) independently assessed the images using a 2-point scale (1 = qualified, 0 = not qualified). The assessment focused on two critical aspects for Gross Tumor Volume (GTV) delineation: the clarity of the tumor-to-normal tissue interface and the veracity of contrast enhancement in tumor invasion risk areas.

### Quantitative evaluation

For quantitative evaluation, two popular evaluating metrics mean absolute error (MAE) and peak signal-to-noise Ratio (PSNR) were used (17, 18). The metrics are expressed as follows (Equations 1, 2):

(1) $$\text{MAE}=\frac{1}{\text{N}}\sum_{\text{i}=1}^{\text{i}=\text{n}}|\text{y}(\text{x})-\text{g}(\text{x})|$$

(2) $$PSNR=10log10\left(\frac{max^{2}}{MSE}\right)$$

(3) $$\text{MSE}=\frac{1}{\text{N}}\sum_{\text{i}=1}^{\text{i}=\text{n}}{\left(y\left(x\right)-g\left(x\right)\right)}^{2}$$

Where N represents the total number of pixels of the CE-MRI and VCE-MRI; $y\left(x\right)$ and $g\left(x\right)$. denote the pixel value of CE-MRI and VCE-MRI, respectively; max is the maximum pixel value of CE-MRI. MSE (Equation 3) is calculated from the square of the original image pixel value minus the generated image pixel.

### Clinical characteristics grouping

Tumor diameter: Tumor diameter was measured on the maximum dimension plane and then grouped into 1 cm intervals from 1 cm to 11 cm. [1,2), [2,3), [3,4), [4,5), [5,6), [6,7), [7,8), [8,9), [9,10), [10,11). Tumor Shape: Tumor morphology was classified as regular or complex based on visual assessment, using specific morphological anchors to ensure reproducibility. Regular was defined as a smooth, continuous tumor margin without deep irregular infiltrative projections. Complex was defined by an irregular margin exhibiting at least one of the following features: multinodular fusion, deep lobulation, or tongue-like projections invading adjacent tissues, resulting in a jagged or markedly uneven tumor-tissue interface (19, 20). T Stage: According to The Eighth Edition AJCC Cancer Staging Manual (21), patients were categorized into four groups: T1, T2, T3, T4. Gender and Age: Gender is categorized into male and female, and age is divided into six groups: under 30, 30-39, 40-49, 50-59, 60-69, and 70 and above.

### Analysis of clinically unacceptable images

Patients were categorized into clinically acceptable and unacceptable sets based on VCE-MRI scores. Comparative analyses of the clinical characteristics of patients in both sets were conducted to investigate the association between image acceptability and clinical characteristics, as well as the underlying reasons for image unacceptability.

### Diagnostic concordance assessment

To evaluate clinical applicability beyond image quality, we assessed the concordance of T-staging between VCE-MRI and real CE-MRI. All images were randomized and independently reviewed by two radiologists blinded to clinical information and image type. The reference standard for staging classification was the Eighth Edition AJCC Cancer Staging Manual (21, 22). Tumors were categorized as T1 through T4 based on the extent of invasion into the nasopharynx, parapharyngeal space, skull base, and intracranial structures. Given that VCE-MRI synthesis focused on primary tumor delineation, our analysis was restricted to T-staging. The concordance rate of T-staging results between CE-MRI and VCE-MRI was calculated.

### Statistical analysis

Statistical analysis was conducted using SPSS 24.0 (SPSS, Chicago, IL, USA). The consistency of radiologists’ rating scores was assessed using Kendall’s coefficient of concordance (W), with values interpreted as follows: W ≤ 0.4 indicating poor consistency, 0.4 < W ≤ 0.8 indicating moderate consistency, W>0.8 indicating excellent consistency (23). One-way ANOVA was used to compare mean VCE-MRI scores across tumor diameter, age, and T-stage groups. Independent samples t-test was employed to assess the significance of mean score in gender and tumor shape groups. Additional comparisons between the unacceptable and the acceptable image sets included MAE, PSNR, tumor diameter, age and mean scores using independent samples t-tests.

To assess the stability of the acceptability rates and stratification criteria, bootstrap resampling (1,000 iterations) was performed on the primary cohort. For each resampled dataset, subgroup acceptance rates and 95% confidence intervals (CIs; percentile method) were recalculated. Overfitting was evaluated by calculating the optimism, defined as the difference in performance between the bootstrap samples and the out-of-bag samples.

## Results

### Patient characteristics

A total of 333 NPC patients met the inclusion criteria (**Figure 1A**). The clinical characteristics of the cohort are summarized in **Table 2**. The median age was 53 years ± 12 [SD] (range: 19 - 84), with 253 (76.0%) male and 80 (24.0%) female patients. The median maximum tumor diameter was 3.4 cm (range: 1.5 - 10cm). According to the Eighth Edition AJCC Cancer Staging Manual (21), the distribution of T stages was: T1 (n=12, 3.6%), T2 (n=102, 30.7%), T3 (n=140, 42.0%), and T4 (n=79, 23.7%).

**Table 2:** Clinical characteristics of patients.

Clinical characters	All patients set	Clinically acceptable VCE-MRI set	Clinically unacceptable VCE-MRI set	*P* value
Count	333	290	43	
Mean Score		4.5 ± 0.3	3.6 ± 0.3	<0.001
M:F ratio	253:80 (76%: 24%)	219:71 (75.5%:24.5%)	34:9 (79.0%:21.0%)	
Age (year)	53 ± 12	53 ± 12	55 ± 11	0.358
T1	12 (3.6%)	12 (4.1%)	0	
T2	102 (30.7)	97 (33.4%)	5 (11.6%)	
T3	140 (42%)	126 (43.4%)	14 (32.6%)	
T4	79 (23.7%)	55 (19.1%)	24 (55.8%)	
Diameter (cm)	3.4 ± 1.2	3.2 ± 1.0	4.6 ± 2.0	<0.001
Shape (regular: complex) ratio	210:123 (63.1%:36.9%)	199:91 (68.6%:31.4%)	11:32 (25.5%:74.5%)	
MAE	46.7 ± 10.2	46.6 ± 9.5	48.4 ± 14.1	0.424
PSNR (dB)	28.7 ± 1.5	28.7 ± 1.5	28.7 ± 2.0	0.967

NA, not applicable; MAE, mean absolute error; PSNR, peak signal-to-noise Ratio.

*P* value indicates whether there are statistically significant differences in clinical characteristics between the acceptable and unacceptable VCE-MRI sets.

### Image evaluation

#### Quantitative evaluation

For the quantitative evaluation of synthesized VCE-MRI, the mean MAE was 46.74 ± 10.17 [SD], and the mean PSNR was 28.7 dB ± 1.53 [SD] (**Table 2**).

#### Assessment result of VCE-MRI

**Figure 2:**
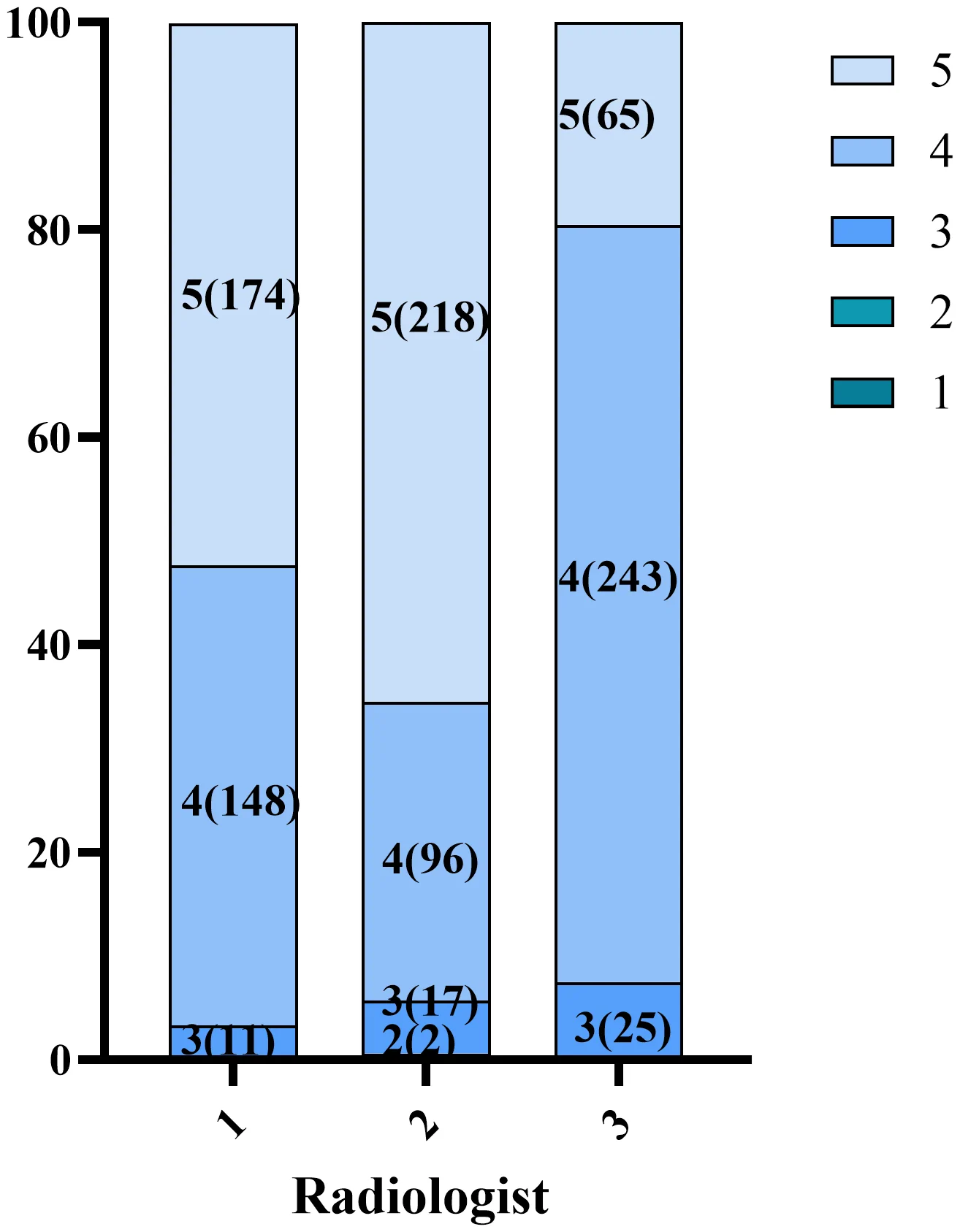
Scores of three radiologists to VCE-MRI.

The scores assigned by three radiologists for 333 VCE-MRI are presented in **Figure 2**. Kendall’s W test was used to evaluate the inter-reader agreement for the three radiologists, resulting in a value of 0.572 (*P* < 0.001), indicating moderate agreement.

Based on the predefined criteria (all three radiologists scoring ≥4), 290 out of 333 VCE-MRI cases (87.1%) were deemed clinically acceptable. The remaining 43 cases (12.9%) were classified as clinically unacceptable.

**Figure 3:**
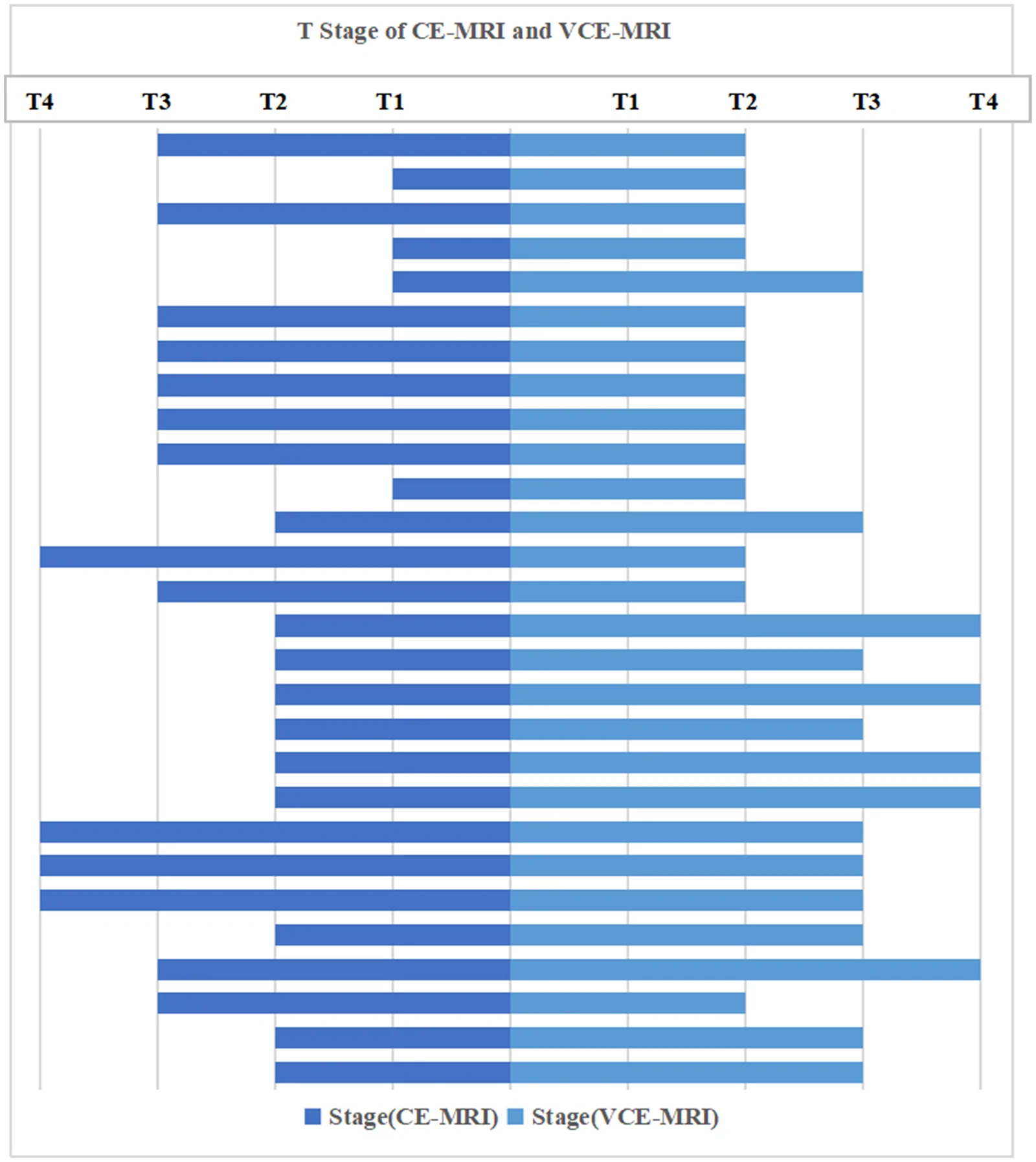
Details of the 28 patients who had different T stages between CE-MRI and VCE-MRI. 15 VCE-MRI obtained advanced T stages compared to actual stages.

Independent T-staging assessment revealed the discordance between VCE-MRI and CE-MRI in 28 patients (8.4%). Notably, VCE-MRI resulted in a more advanced T-stage in 15 of these 28 cases (**Figure 3**).

We analyzed clinical acceptability rates, stratified by scanner vendor and field strength. For the three vendors, the rates were 89.3% (75/84) for SIEMENS, 85.6% (125/146) for GE, and 87.4% (90/103) for Philips (p = 0.66). When grouped by field strength, the acceptability rate was 89.3% (75/84) at 1.5T and 86.3% (215/249) at 3T (p = 0.40), showed in **Table 3**.

**Table 3:** Clinical acceptability and clinical characteristic grouping.

Clinical Characteristic Groups	No. of patients (ratio)	clinical acceptability (ratio)	Average score	*P* Value
Diameter (cm)				
[1,2)	15 (4.5%)	14 (93.3%)	4.4 ± 0.4	
[2,3)	117 (35.1%)	110 (94.1%)	4.5 ± 0.4	
[3,4)	124 (37.2%)	112 (90.3%)	4.4 ± 0.4	
[4,5)	46 (13.8%)	39 (84.8%)	4.3 ± 0.5	
[5,6)	16 (4.8%)	9 (56.2%)	4.2 ± 0.5	
[6,7)	6 (1.8%)	3 (50.0%)	4.2 ± 0.4	
[7,8)	5 (1.5%)	3 (60.0%)	4.2 ± 0.4	
[8,9)	3 (0.9%)	0 (0)	3.7	
[9,10)	NA	NA	NA	
[10,11)	1(0.3%)	0 (0)	3.7	0.001
Shape				
regular	210 (63.1%)	199 (94.8%)	4.5 ± 0.4	
complex	123 (36.9%)	91 (74.0%)	4.2 ± 0.5	<0.001
Stage				
T1	12 (3.6%)	12 (100%)	4.6 ± 0.4	
T2	102 (30.7%)	97 (95.1%)	4.5 ± 0.4	
T3	140 (42%)	130 (90.0%)	4.4 ± 0.4	
T4	79 (23.7%)	55 (69.6%)	4.2 ± 0.5	<0.001
Gender				
Male	253 (76%)	219 (86.6%)	4.4 ± 0.4	
Female	80 (24%)	71 (88.7%)	4.4 ± 0.4	0.773
Age (year)				
<30	6 (1.8%)	0 (0)	4.4 ± 0.3	
30-39	43 (12.9%)	5 (11.6%)	4.4 ± 0.4	
40-49	92 (27.6%)	10 (10.9%)	4.4 ± 0.4	
50-59	104 (31.3%)	18 (17.3%)	4.4 ± 0.5	
60-69	61 (18.4%)	4 (6.6%)	4.4 ± 0.4	
≥70	27 (8%)	6 (22.2%)	4.2 ± 0.5	0.327
Vendor				
SIMENS	84 (25.2%)	75 (89.3%)		
GE	146 (43.8%)	125 (85.6%)		
Philips	103 (30.9%)	90 (87.4%)		0.66
Field Strength				
1.5T	84 (25.2%)	75 (89.3%)		
3T	249 (74.8%)	215 (86.3%)		0.40

The distribution of patients is detailed across various clinical characteristics groups, including tumor diameter, tumor shape, T stage, gender, and age groups, and the mean scores, the number, and the proportion of clinically acceptable cases within each group. The clinical acceptability across different field strengths and vendors are also shown. The *P* value indicates whether the mean scores within the respective clinical characteristic groups exhibit statistically significant differences.

#### Clinical acceptability and clinical characteristic grouping

The association between clinical characteristics and VCE-MRI acceptability is detailed in **Table 3**.

Tumor diameter: The acceptability of VCE-MRI decreased with increasing maximum tumor diameter, evident for diameter larger than 5cm, with 56.2% in the [5, 6) cm group, 50.0% in the [6, 7) cm group, and 0 in both the [8, 9) cm and [10, 11) cm group. The mean score decreased with increasing tumor diameter (*P* = 0.001).

Tumor shape: The clinical acceptability of regular and complex groups were 94.8% and 74.0%, respectively. The mean scores of regular and complex tumor shape groups were 4.5 ± 0.4 and 4.2 ± 0.5 (*P* < 0.05) (**Table 3**).

T stage: A significant inverse correlation was observed between T-stage and VCE-MRI acceptability. The clinical acceptability decreased significantly with T-stage: T1: 100% (12/12), T2: 95.1% (97/102), T3: 90.0% (130/140), T4: 69.6% (55/79) (*P* < 0.001). Mean scores decreased progressively with higher T-stage, 4.6 ± 0.4 for T1, 4.5 ± 0.4 for T2, 4.4 ± 0.4 for T3, and 4.2 ± 0.5 for T4 (*P* < 0.001).

Gender and age: The mean scores for male and female groups were both 4.4 ± 0.4 (*P* = 0.773). The distribution of scores by age group is presented in **Table 3**, with a *P*-value of 0.327, indicating that neither gender nor age demonstrated a significant association with VCE-MRI acceptability.

Target delineation evaluation: 93.4% of CE-MRI and 92.8% of VCE-MRI images were judged qualified by the radiation oncologists(p=0.768). The remaining cases were deemed unqualified, primarily owing to suboptimal image quality and indistinct tumor interfaces. These limitations were largely attributed to the use of older MRI scanners and the highly infiltrative nature of certain tumors, which hindered precise margin identification.

#### Analysis of clinically unacceptable VCE-MRI

Out of the 333 patients reviewed, 43 were clinically unacceptable and 290 were acceptable. The unacceptable group had a higher proportion of patients with advanced T-stage (T3/T4: 88.4% vs 62.5%), larger mean tumor diameter (4.6 ± 2.0 cm vs 3.2 ± 1.0 cm, *P* < 0.001), and complex tumor shape (74.5% vs 31.4%). No significant differences in mean MAE (*P* = 0.424) and mean PSNR (*P* = 0.967) of the two sets was found.

**Figure 4:**
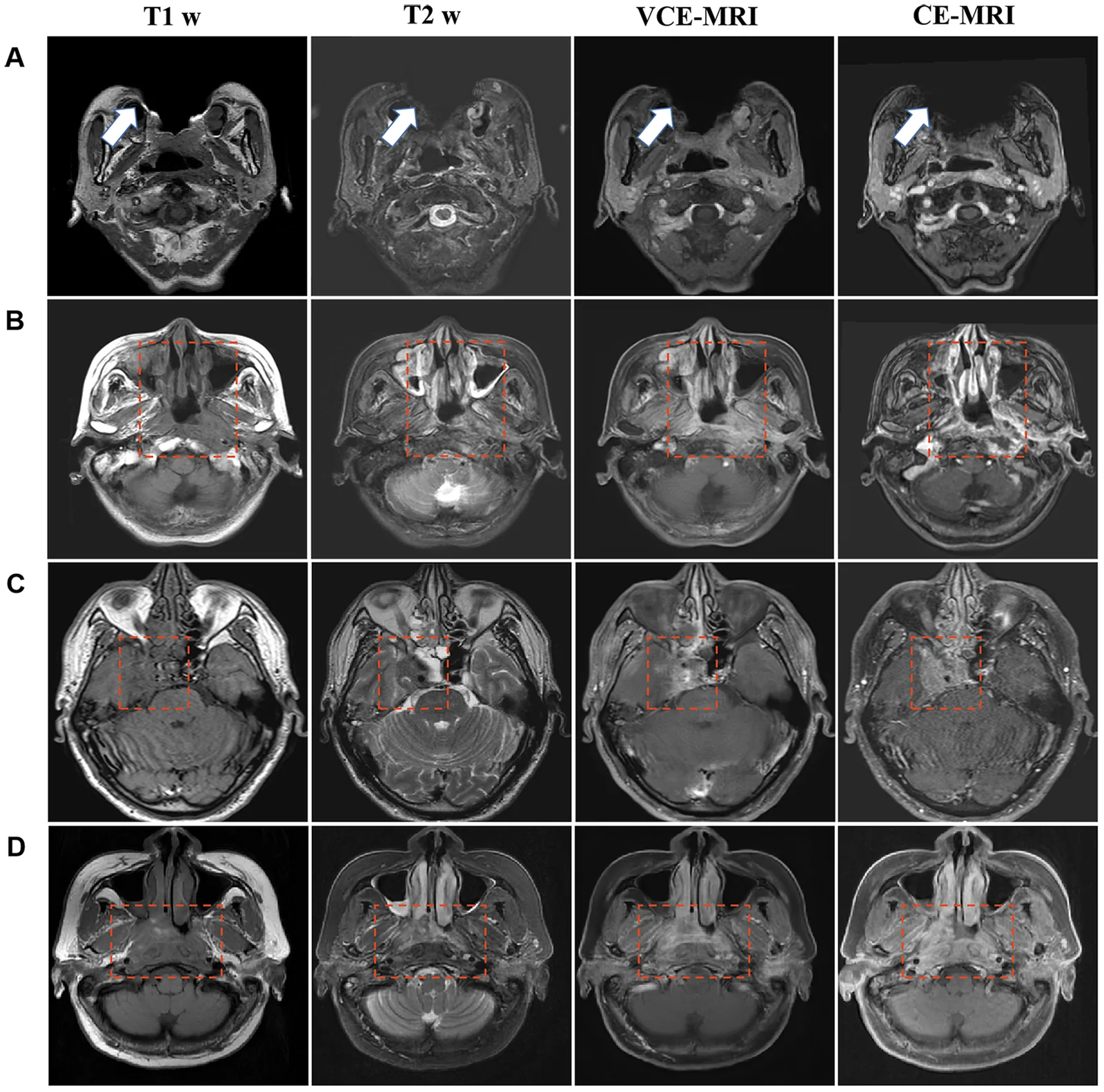
Example NPC MRI from the study. From left to right: T1-weighted images, T2-weighted images, virtual contrast-enhanced MRI(VCE-MRI), and contrast-enhanced MRI(CE-MRI). **(A)** The presence of metal artifacts in the oral cavity. **(B)** Volume effects resulted in image blur, affecting not just a single slice. **(C)** There were changes in the tumor extent visible in the VCE-MRI. **(D)** The boundaries of the tumor and surrounding structures were indistinct.

Review of the 43 unacceptable cases, we identified the primary reason for rejection (some cases had multiple issues), 13 due to image artifacts (including metal, motion, volume effects), 20 due to unclear structural boundaries (tumor boundaries or surrounding structures), and 10 due to tumor changes (tumor extent, margin, tumor density). Representative examples illustrating these issues are provided in **Figure 4**, including metal artifacts (A), volume effects (B), tumor extent change (C), and unclear boundaries (D).

**Figure 5:**
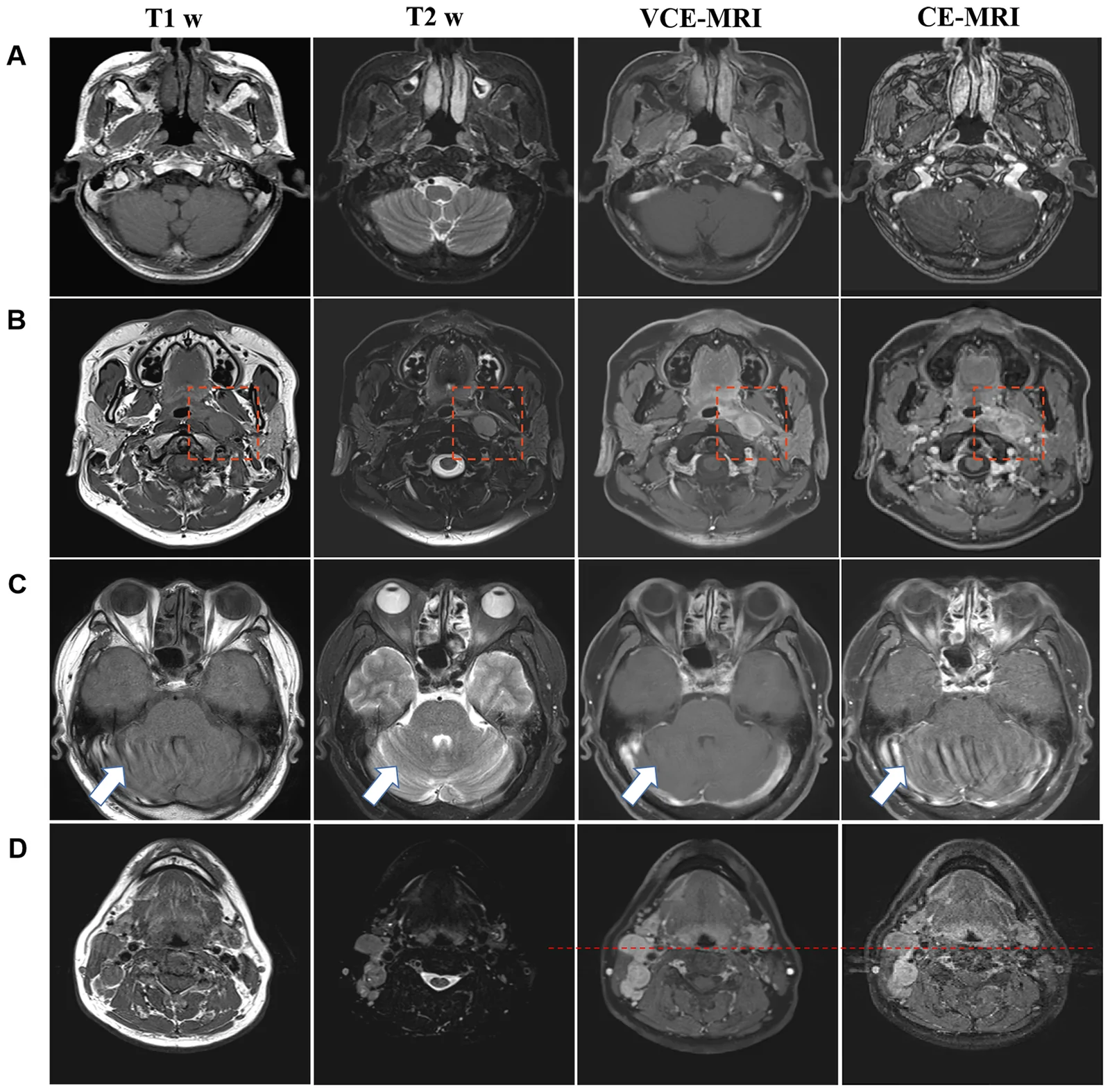
Example NPC MRI from the study. From left to right: T1-weighted images, T2-weighted images, virtual contrast-enhanced MRI(VCE-MRI) and contrast-enhanced MRI(CE-MRI). **(A)** The noise in the VCE-MRI images was significantly reduced compared to the CE-MRI. **(B)** The noise reduction in the VCE-MRI images was accompanied by clearer boundaries of the tumor and surrounding structures. **(C)** The VCE-MRI notably reduced the artifacts in the brain area. **(D)** There was a marked displacement in the pharynx and lymph nodes on the VCE-MRI compared with input images and CE-MRI.

In a subset of cases, VCE-MRI appeared to mitigate certain limitations present in the input or even reference CE-MRI images. Specifically, reductions in image noise and artifacts were observed in some VCE-MRI studies compared to their corresponding CE-MRI (**Figures 5A–C**). However, motion-related structural displacement, primarily affecting the pharynx and lymph nodes, was noted as a challenge in some VCE-MRI studies (**Figure 5D**).

#### Internal validation

Bootstrap validation (1,000 iterations) confirmed the stability of the stratification criteria. The acceptance rates and their 95% CIs were: T1/T2, 95.6% (95% CI: 91.2%-99.1%); T3/T4 with regular shapes, 94.2% (95% CI: 89.8%-98.1%); and T3/T4 with complex shapes, 68.4% (95% CI: 59.0%-76.8%). The negligible optimism (<0.25%) across all subgroups indicated minimal overfitting. Furthermore, the non-overlapping 95% CIs between the high-acceptability subgroups (T1/T2, T3/T4 regular) and the low-acceptability subgroup (T3/T4 complex) confirmed the robustness of the shape-based stratification.

## Discussion

Deep learning methods for generating virtual contrast-enhanced MRI (VCE-MRI) from non-contrast sequences offer substantial potential to reduce reliance on GBCAs and associated safety concerns (10–13, 24, 25). Generative AI models are conventionally evaluated via pixel-wise comparisons, relying on metrics such as MSE, RMSE, PSNR, and SSIM (26). However, these technical parameters possess inherent limitations. Khawaled et al. recently delineated critical flaws in applying reference metrics like PSNR and SSIM to synthetic medical images, notably their inability to detect localized structural errors and their poor correlation with human perceptual quality (27).

This disjunction between technical fidelity and diagnostic utility is particularly evident in our cohort. Although synthesized images might achieve acceptable global technical metrics, the clinical acceptability rate plummeted to 68.4% for T3/T4 tumors with complex morphologies. In these instances, subtle boundary blurring or morphological distortions—artifacts that are mathematically negligible yet critically compromise T-staging—rendered the images diagnostically unacceptable. Consequently, emerging literature increasingly emphasizes that evaluation paradigms should transcend mere technical quality parameters to prioritize real-world clinical efficacy (28). Recognizing this imperative, our multi-institutional study assessed the clinical applicability of a deep learning-based VCE-MRI technique (MMgSN-Net) in 333 patients with nasopharyngeal carcinoma (NPC), and proposed stratification criteria based on expert clinicians’ assessment.

Our findings demonstrate that tumor characteristics, T-stage, maximum diameter, and shape complexity are the primary factors influencing the clinical acceptability of VCE-MRI, whereas patient age and gender showed no significant association. The decline in VCE-MRI quality with increasing T-stage, larger tumor size, and irregular morphology is consistent with the biological complexities these features pose. Higher T-stage tumors tend to infiltrate more deeply and involve more anatomically intricate regions (e.g., parapharyngeal space, intracranial extension, cranial nerves) (22, 29, 30). Similarly, larger tumors and those with spiculated or irregular margins often exhibit greater heterogeneity and poorly defined boundaries (22, 29–31), which complicates the extraction and synthesis of enhancement features by deep learning models (11, 32). These findings align with previous radiomics studies demonstrating that tumor volume (31, 33) and shape complexity (19) significantly influence a wide range of extracted features, further supporting our combined stratification criteria.

Artifacts were the direct cause of clinical unacceptability in 13 cases (30.2% of unacceptable cases), underscoring previous research by Leijenaar et al. and Gere et al. that image artifacts can impair features essential for analysis (34, 35). Our study involved 13 MRI scanners (**Table 1**), reflects the diversity of scanning protocols but also introduces variability. Scanning parameters will influence the texture features of MRI during the image acquisition phase, like magnetic field strength, repetition time (TR), echo time (TE), scanning bandwidth (SBW), particularly for spatial resolution (36–38). Suboptimal parameter settings found in our study, such as overly short T1-weighted TR (10ms) will reduce SNR and resolution, or excessively long T2-weighted TR (12154ms) will obscure tissue details, these contributed to low-quality inputs in some cases, ultimately degrading the accuracy of VCE-MRI. Roy Sudipta, et al. and K Lafata et al. discovered the performance of radiomic models is directly affected by SNR (33, 39). Our result confirmed that low resolution and low SNR of input images were factors in some clinically unacceptable cases (a low average SNR was 13.2 for six affected cases). Notably, our analysis revealed no significant differences in clinical acceptability among vendors or between 1.5T and 3T field strengths. While this may be attributed to considerable variability in scanning parameters across institutions, it also underscores the model’s robustness and clinical applicability in real-world clinical settings. Future studies could further investigate the impact of standardized scanning parameters on synthesis quality to optimize performance in specific clinical scenarios.

Interestingly, we observed that in certain cases, VCE-MRI synthesis appeared to improve upon limitations seen in both input images and even the CE-MRI, such as by reducing noise and partially mitigating artifacts (**Figures 5A–C**). This suggests a potential secondary role for VCE-MRI, not only as a contrast surrogate but also as a post-processing enhancement technique under suboptimal acquisition conditions. However, VCE-MRI remains susceptible to motion artifacts, particularly those caused by swallowing, which lead to misalignment in pharyngeal or lymph node regions (**Figure 5D**). This highlights the current limitation in handling motion artifacts.

Given the strong associations between tumor T-stage and tumor morphology with VCE-MRI quality, we proposed a pragmatic stratification strategy (**Figure 1B**). MMgSN-Net-generated VCE-MRI demonstrated excellent clinical acceptability (≥95%) in patients with early-stage (T1/T2) NPC, and in advanced-stage (T3/T4) NPC with regular tumor shape. Approximately 67.3% of our cohort supports the deep learning based VCE-MRI, indicating a potential to replace the conventional CE-MRI and consequently lead to a significant reduction of GBCA exposure. By contrast, caution is warranted when applying VCE-MRI to T3/T4 tumors with complex shapes, where acceptability dropped to 73.0%. The indistinct boundaries and intricate infiltration patterns characteristic of these lesions likely increase the risk of synthesis errors, potentially affecting critical tasks like staging and target delineation. For these patients, conventional CE-MRI remains preferred option.

Our study has several limitations. As a retrospective study, there is an inherent risk of data drift; differences in equipment, protocols, and patient cohorts between training and validation may affect performance evaluation. Furthermore, image quality interpretation relied on visual assessment by three radiologists using a clinical acceptability index. While this reflects clinical practice, visual assessment remains inherently subjective. To mitigate this bias, we calculated the Kendall’s coefficient of concordance (W) for inter-observer agreement. Furthermore, the lack of significant impact from age and gender may be influenced by cohort size and distribution of our study cohort. Therefore, we cannot preclude the possibility of underlying biological differences, such as variations in tissue fat composition (40). The morphological assessment in this study relies on visual evaluation, as tumor segmentation was not performed, extracting objective 3D radiomics shape features (e.g., sphericity and compactness) was not feasible. Future studies will incorporate tumor segmentation on CE-MRI and VCE-MRI to enable quantitative morphological analysis and further validate these findings. The potential effect of N-stage or prior treatment history on image quality and model performance was not investigated in this study. Finally, VCE-MRI remains vulnerable to motion artifacts, particularly from swallowing, highlighting the need for improved patient immobilization techniques or integration of motion correction strategies during VCE-MRI synthesis.

In conclusion, this multi-institutional study provides robust evidence supporting the clinical utility of MMgSN-Net-based VCE-MRI for a well-defined group of NPC patients. By establishing stratification criteria based on patients’ clinical characteristics, we identified T1/T2-stage NPC patients and T3/T4-stage patients with regular tumor shapes as suitable candidates, achieving high clinical acceptability rates of over 95.0%. Implementing this stratification could potentially reduce GBCA administration in approximately two-thirds of eligible NPC patients. For patients with advanced T-stage and complex tumor shapes, VCE-MRI quality is currently less reliable, warranting continued use of conventional CE-MRI. Future works should focus on refining deep learning models to better handle complex tumor morphologies and image artifacts, improving input image robustness, and prospectively validating the clinical utility of VCE-MRI within the defined stratification framework for specific diagnostic and therapeutic tasks.

## Data Availability

Research data are stored in an institutional repository and will be shared upon request to the corresponding author.
